# Characterizing Tetraploid Populations of *Actinidia chinensis* for Kiwifruit Genetic Improvement

**DOI:** 10.3390/plants11091154

**Published:** 2022-04-24

**Authors:** Zhi Wang, Guangming Hu, Zuozhou Li, Caihong Zhong, Xiaohong Yao

**Affiliations:** 1 Institute of Fruit and Tea, Hubei Academy of Agricultural Sciences, Wuhan 430064, China; kiwiwz@foxmail.com; 2 CAS Key Laboratory of Plant Germplasm Enhancement and Speciality Agriculture, Wuhan Botanical Garden, the Chinese Academy of Sciences, Wuhan 430074, China; wangyi_guangming@163.com (G.H.); lizz@wbgcas.cn (Z.L.); 3 College of Life Sciences, University of Chinese Academy of Sciences, Beijing 100049, China

**Keywords:** genetic diversity, structure, *A. chinensis*, SSR, ENM

## Abstract

Understanding genetic diversity and structure in natural populations and their suitable habitat response to environmental changes is critical for the protection and utilization of germplasm resources. We evaluated the genetic diversity and structure of 24 *A. chinensis* populations using simple sequence repeat (SSR) molecular markers. The potential suitable distribution of tetraploid *A. chinensis* estimated under the current climate and predicted for the future climate was generated with ecological niche modeling (ENM). The results indicated that the polyploid populations of *A.* *chinensis* have high levels of genetic diversity and that there are distinct eastern and western genetic clusters. The population structure of *A. chinensis* can be explained by an isolation-by-distance model. The results also revealed that potentially suitable areas of tetraploids will likely be gradually lost and the habitat will likely be increasingly fragmented in the future. This study provides an extensive overview of tetraploid *A. chinensis* across its distribution range, contributing to a better understanding of its germplasm resources. These results can also provide the scientific basis for the protection and sustainable utilization of kiwifruit wild resources.

## 1. Introduction

Kiwifruit is a perennial, dioecious economic fruit that is native to China. It is one of the best examples of the successful domestication and commercialization of crops in the early 20th century [1]. Kiwifruit is not only rich in vitamin C and minerals but also has medicinal and ornamental value [2,3]. In recent years, the value of kiwifruit in the international market is becoming more and more prominent [4]. The total output of cultivated kiwifruit in the world is about 3 million tons, of which China accounts for about half [1]. The chromosome ploidy of kiwifruit is complex, with intertaxon ploidy variation having so far been detected in at least 13 *Actinidia* species (2n = 2x = 58, 2x, 3x, 4x, 5x, 6x, 7x, 8x, etc.). Previous studies have shown that *Actinidia* has experienced at least eight interspecific hybridization events in the process of evolution [5]. With the rapid evolution of kiwifruit backbone lineages caused by frequent interspecific hybridization and the formation of hybrid populations derived from these lineages, reticulate species is an important mechanism for the maintenance of biodiversity [6]. *Actinidia chinensis* is the species with the highest domestication level and the greatest economic benefit among the species of *Actinidia* [7]. Although the germplasm resources are abundant, their genetic diversity is also being threatened and challenged [8]. The effective evaluation of germplasm will be needed to ensure the sustainable and healthy development of the kiwifruit industry globally [9].

In recent years, the number of studies on the genetic diversity of *A. chinensis* has been increasing, providing us with a better understanding of this fruit crop [10,11]. Crossbreeding between different chromosome ploidy levels of *A. chinensis* can produce fertile offspring, which is often accompanied by excellent traits for crop improvement [5,9]. The most obvious example is that tetraploid varieties have better resistance to canker than diploid varieties [12]. Tetraploids also yield bigger fruits and better quality, and also have a better adaptability and faster growth speed [13]. Among the current cultivars, tetraploid varieties are most prevalent [9]; therefore, it is desirable to evaluate the genetic variation of tetraploid individuals from natural populations. However, population genetic studies of tetraploid *A. chinensis* are scarce, and the origin of tetraploid *A. chinensis* remains unclear [8]. In previous studies, researchers either focused on the analysis of genetic diversity and the population structure of *A. chinensis* in limited regions [14] or focused on diploid populations [8]. Neither the population structure nor genetic diversity of tetraploid *A. chinensis* have been reported.

Simple sequence repeat (SSR) markers, which are based on genome sequences, are easy to use, relatively low in cost, and have high polymorphism and extensive genetic information. It is the most widely used type of DNA molecular marker to characterize genetic germplasms, with many alleles at each locus [15]. Previous studies have indicated that climate is the main environmental factor affecting species distribution at a regional scale [16]. Ecological niche modeling (ENM) has played an important role in studying the effects of climate change on species distribution. The Maxent model has been shown to have the best predictive accuracy and stability [17,18] for revealing the effects of global climate change on species distribution [19]. In this study, we investigated the genetic diversity of natural populations of *A. chinensis* dominated by tetraploid individuals based on 40 microsatellite markers. In addition, 52 distribution points of tetraploid *A. chinensis* were used for niche simulation. The objectives of this study were to (1) evaluate the genetic diversity of the tetraploid component of *A. chinensis*; (2) describe the tetraploid population structure; (3) predict the potential suitable distribution for tetraploid *A. chinensis* in both current and future climate; and (4) provide breeding and conservation strategies for kiwifruit germplasm.

## 2. Results

### 2.1. Genetic Diversity of A. chinensis Populations

**Figure 1:**
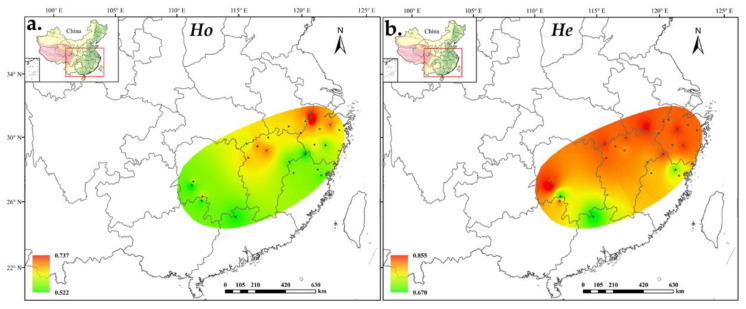
Distribution of *A. chinensis* population diversity based on (**a**) observed heterozygosity and (**b**) expected heterozygosity.

We detected 758 alleles for 24 *A. chinensis* populations. The average number of alleles per locus was 18.9, of which, the minimum number of alleles detected at each locus was 9 (at UDK96-009 locus) and the maximum number was 28 (at UDK96-034 locus). The polymorphism information content (PIC) varied from 0.213 (UDK96-028) to 0.924 (UDK96-019), with an average of 0.808 (Table S1). Most of the alleles were shared by the diploid and tetraploid populations, whereas 239 alleles were unique for the tetraploid populations and only 6 alleles were unique for the diploid populations (Table S2). At the population level, the effective number of alleles (*N*e) ranged from 3.037 in ZG to 6.437 in ZY, averaging 5.124 alleles per population. The inbreeding coefficients (*F*is) were all greater than zero, ranging from 0.080 (LA) to 0.389 (DN), with an average of 0.024 (Table 1). The expected heterozygosity (*H*e) as estimated using GENODIVE ranged from 0.670 (NL) to 0.855 (DN), whereas the *H*e estimated using POLYGENE ranged from 0.636 (ZY) to 0.795 (DN). However, the value of observed heterozygosity (*H*o) calculated using POLYGENE ranged from 0.681 (NL) to 0.808 (LA), and the same trend has been observed in GENODIVE. The observed gene heterozygosity was lower than the expected gene heterozygosity. On average, the genetic diversity of the tetraploid population was higher than that of the diploid population, although it was different in some populations such as LA and PN. (Figure 1).

### 2.2. Genetic Structure and Differentiation of A. chinensis

**Figure 2:**
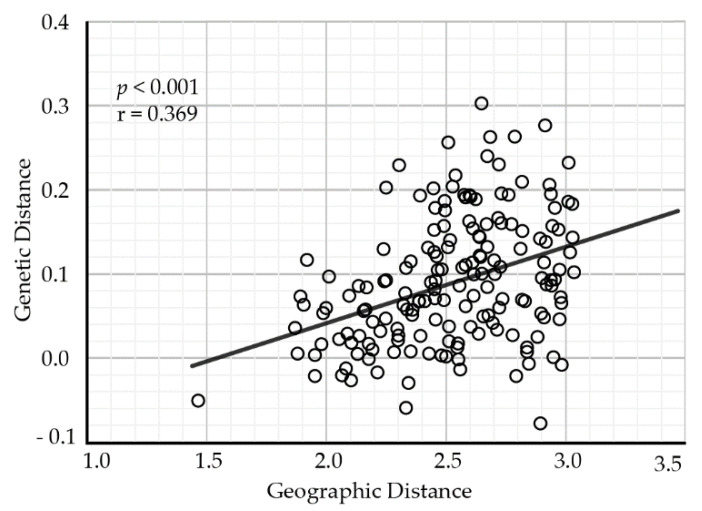
The relationship between genetic differentiation and geographical distance for *A. chinensis* populations.

The pairwise comparisons of genetic differentiation between populations showed that *G*_ST_ ranged from 0.0002 between populations XN and TT to 0.176 between populations ZG and NL. Some populations, for example JX, QM, and XN, were less divergent from the other populations (Table S3). The results of the Mantel test revealed that geographical distance (the natural logarithm-transformed) was positively related to genetic distance (as measured by Slatkin’s linearized *F*_ST_) among populations (Mantel test: r = 0.369, *p* < 0.001), indicating the presence of an isolation-by-distance effect (Figure 2).

The purpose of the analysis of molecular variance (AMOVA) was to see if there was any genetic variation across populations as well as within populations. According to our results (Table 2), the AMOVA revealed that the genetic variation is mostly within populations (90.99%), whereas only 9.01% of variance was attributed to among-population differentiation. When the ploidy level was analyzed, only 3.17% of the genetic variation was distributed among diploids and tetraploids, whereas 96.83% of the total variation occurred within ploidy types.

**Figure 3:**
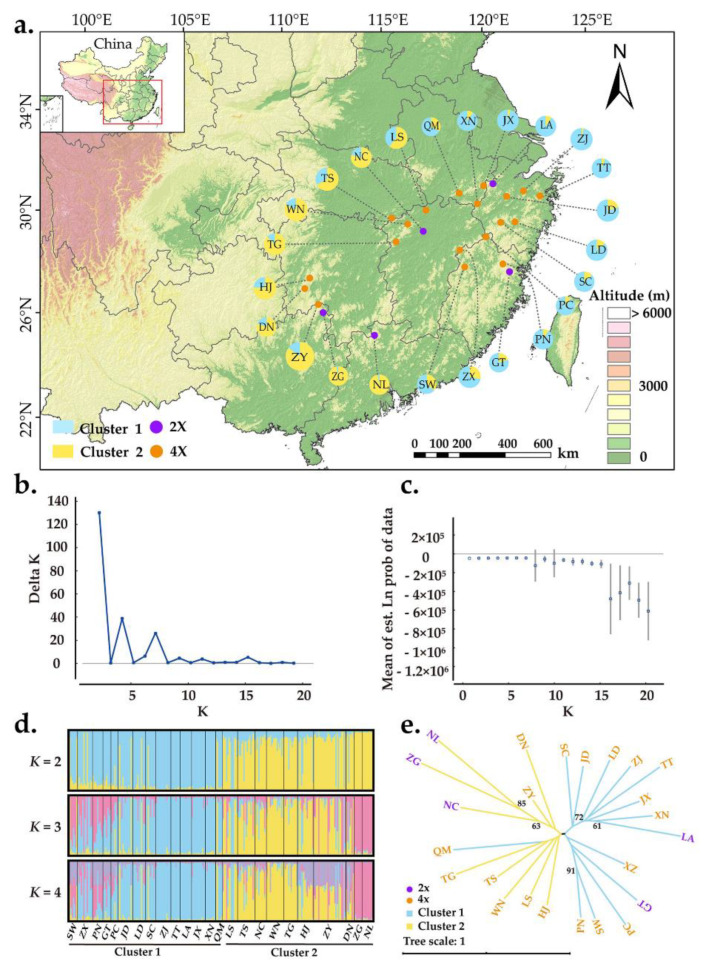
Population genetic structure of *A. chinensis*. (**a**) Geographic distribution of the 24 populations. Each population is color-coded in different clusters according to the results of STRUCTURE analysis. The purple and orange dots represent diploid and tetraploid populations, respectively; (**b**) the ΔK values are presented for *K* = 1–20; (**c**) the likelihood L(*K*) values presented for *K* = 1–20; (**d**) histogram of the Bayesian assignment for 263 individuals in 24 populations of *A. chinensis*. STRUCTURE plots are presented for *K* = 2 to *K* = 4, respectively. Each vertical bar represents one individual and the capital letters on the abscissa represent different populations; (**e**) neighbor-joining tree based on genetic distances for 24 natural populations of *A. chinensis.* The robustness of each node was evaluated by 1000 bootstrap replicate.

The Bayesian assignment revealed that *K* = 2 was the best value when LnP(K) was found to increase and ΔK was maximized (Figure 3b,c). This result suggested that there are two distinct genetic clusters: cluster 1 (eastern population) and cluster 2 (western population) (Figure 3a). Although the two clusters were relatively easy to distinguish, more individuals were shared between them (Figure 3d). As the *K* value increased, more and more individuals were found to have mixed ancestry from multiple genetic clusters (Figure 3d). Geographically, individuals sampled from the same location did not fully cluster together (Figure 3a). However, the populations in cluster 1 were generally distributed in eastern China, whereas populations from cluster 2 were distributed westward. The genetic relationships among *A. chinensis* individuals were further explored with principal coordinate analysis (PCoA), and the results were generally consistent with those of STRUCTURE (Figure S1). Furthermore, a neighbor-joining tree was constructed with genetic distances where populations were also divided into two groups, consistent with results of both STRUCTURE and PCoA. Diploid and tetraploid populations were mixed in both genetic groups.

### 2.3. Environmental Niche of Tetraploid A. chinensis

**Figure 4:**
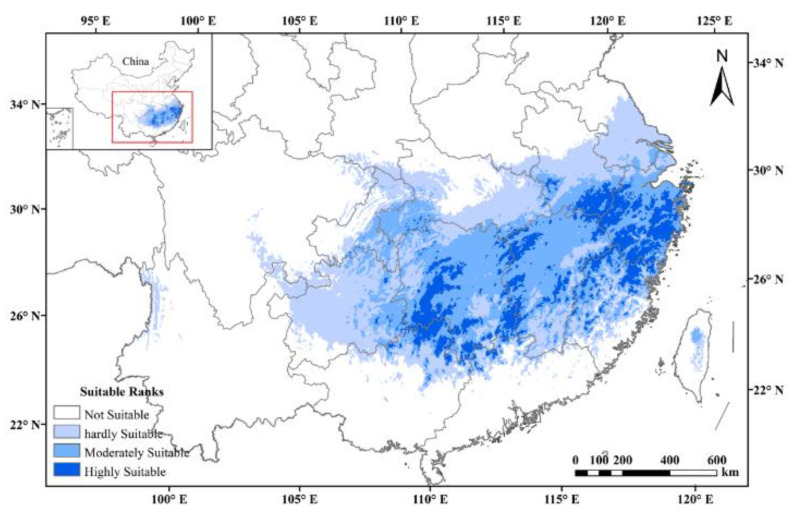
Potential distributions of tetraploid *A. chinensis* under current climatic scenarios in China.

The AUC values of ENM for tetraploids with each climate scenario were high (i.e., a greater than 0.9), indicating that all models performed well in predicting the suitable habitat under all climate scenarios. Under the current climatic scenarios, the potentially suitable area was a good representation of the actual distribution of tetraploid *A. chinensis*. The results of prediction and reclassification showed that the potentially suitable area of tetraploid *A. chinensis* accounted for 11.3% of the total land area of China. In addition, the suitable habitats could be subdivided into hardly suitable habitats, moderately suitable habitats, and highly suitable habitats, and they accounted for 46.6, 38.2, and 15.5 % of the total suitable area, respectively (Figure 4).

**Figure 5:**
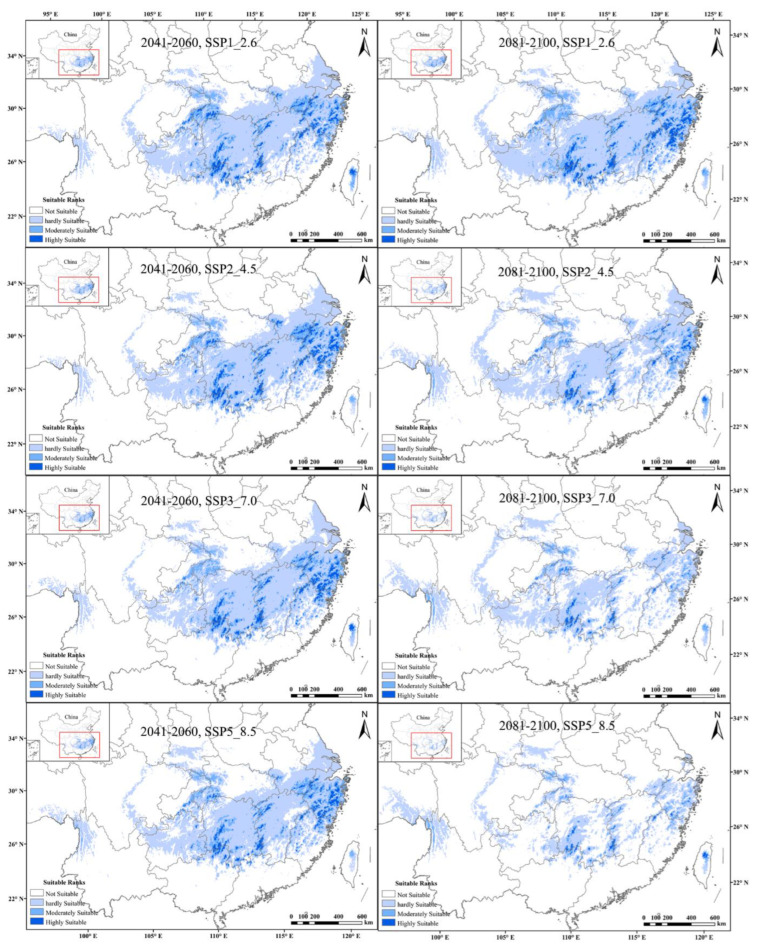
Comprehensive habitat suitability of tetraploid *A. chinensis* under different future scenarios in China.

From the predictions of future global warming scenarios, it was found that the potential distribution area of tetraploid *A. chinensis* decreased substantially under eight different future climate scenarios. The tetraploids’ highly suitable habitats were predicted to decrease by up to about 95.3% under the 2081–2100, SSP5_8.5 scenario, the highest level of the greenhouse gas emission scenarios. Additionally, the moderately and hardly suitable distribution of tetraploid *A. chinensis* showed the same decreasing trend. Additionally, under the 2081–2100, SSP5_8.5 scenario, the reduction rate of tetraploids’ moderately and hardly suitable habitats was found to be the highest, with a total of 50.8% (Figure 5). In conclusion, with the intensification of global climate change, the potentially suitable area of tetraploid *A. chinensis* will be gradually lost, and their habitat will be increasingly fragmented.

## 3. Discussion

### 3.1. Genetic Diversity of Diploids and Tetraploids

Our results reveal the existence of the high genetic diversity of tetraploid *A. chinensis* in subtropical China. The average PIC of the tetraploid populations in this study was also greater than the average of the diploid populations. This is consistent with the results of Wang et al. [14], where the average PIC of wild *A. chinensis* populations from several hexaploid populations in the Qinling Mountains was higher than the values of present tetraploid populations. These results imply that polyploid populations generally have a higher level of genetic diversity compared to diploid populations. The same evidence can also be found in our *H*o or *H*e values of diploids and tetraploids in this study.

In general, the level of genetic diversity possessed by a species reflects its prepared evolutionary potential. Therefore, species with low levels of genetic diversity are more likely to become extinct [20]. During the process of production and cultivation, the comprehensive performance of tetraploid kiwifruit is often better than that of diploid germplasms, especially in stress resistance. For example, tetraploid varieties have stronger resistance to PSA (*Pseudomonas syringae* pv. *actinidiae*) than diploid varieties, which has been observed in many orchards.

### 3.2. Population Genetic Structure and Differentiation

In this study, *A. chinensis* individuals formed two genetic groups in both principal coordinate analysis (PCoA) and Bayesian model-based clustering (Figure S1 and Figure 3d). In addition, the clustering results of STRUCTURE and PCoA were corroborated by the topology of a neighbor-joining tree (Figure 3e). Although some individuals were shared between these two clusters, it is still easy to distinguish their east/west pattern of geographical distribution (Figure 3a). In addition, the Mantel test revealed a positive correlation between genetic divergence (as Slatkin’s linearized *F*_ST_) and geographical distance (Figure 2), suggesting that genetic differentiation in tetraploid *A. chinensis* followed a pattern of isolation by distance. This result is consistent with the previous IBD analysis of *A. chinensis* diploid populations [8], indicating an isolation-by-distance effect. The above results suggest that physical barriers play an additional role in shaping patterns of gene flow between clusters [21], although the genetic differentiation between these two clusters is low (*F*_ST_ = 0.025) (Table 2). The differentiation between clusters 1 and 2 could be explained by climatic and geological changes since the Pliocene, which led to the fragmentation of the habitat of *A. chinensis* and the development of a geographical barrier, as revealed in our studies [22].

In the present study, of the total genetic variation partitioned, 9.01% was attributed to the differences among populations, and 90.99% to the differences among individuals within populations, in agreement with the findings of previous studies on *A. chinensis* [8,14]. The low level of genetic differentiation among populations indicated that gene flow among populations was not limited. The fruit of *A. chinensis* is a desirable food source for frugivory animals. Additionally, the seeds of *A. chinensis* can germinate readily upon maturation and are potentially capable of establishing a new population. Thus, high levels of gene flow among *A. chinensis* population are expected.

### 3.3. Occurrence of Tetraploids in A. chinensis

Polyploidy is widely distributed in plants, and polyploidization is regarded as a major force driving plant evolution and speciation [23,24,25]. Polyploid plants often originate from diploid ancestors, so they usually exhibit increased vigor and competitiveness [26,27] and show a preference for distinct habitats with niche expansion [28,29,30]. This study is the first report of tetraploid population genetic diversity and structure in *A. chinensis.* Although previous studies [22,31] have also involved a few tetraploidy individuals, none have been able to focus on tetraploidy populations. Tetraploid *A. chinensis* is generally considered to be an autopolyploid, and diploid *A. chinensis* was one of its ancestors [32,33]. All tetraploid populations in this study did not form a single cluster in the STRUCTURE or PCA analyses. However, the tetraploid populations always clustered into the same groups as geographically adjacent diploid populations. This suggests that polyploid populations likely originated polyphyletically from their neighboring diploid populations and coexisted with their diploid parents within a certain geographic range. A similar inference has also been found in previous studies of *Galax urceolata* [34] and *G. pentaphyllum* [35]. More definitive assessment as to whether polyploidization in *A. chinensis* arose once or multiple times will require other data such as high-throughput DNA sequence methods.

### 3.4. Implications for Conservation and Utilization

*A. chinensis* is listed in the list of the national key protected wild plants. From a conservation perspective, genetic diversity estimates can be used in making decisions about the management of extant populations of endangered species. In the present study, the high level of genetic diversity maintained within wild populations of *A. chinensis* is encouraging. However, the result of the ENM showed significant decreases in the area of the potential distribution of tetraploid *A. chinensis* under various future climate scenarios. This suggests that climate change will shrink the potential suitable habitat of tetraploid *A. chinensis* under future different emission scenarios. This mainly results from changes in the distribution of temperature and precipitation, which directly affects the boundaries and trends of plant growth [36]. With climatic change in the future, the distribution area of tetraploid *A. chinensis* will tend to migrate to high elevations, and its habitat will be more fragmented. Under these scenarios, there will be more pressure on the conservation and management of tetraploid *A. chinensis* resources in the future.

The high genetic diversity observed in tetraploid *A. chinensis* populations suggests their great potential for kiwifruit breeding. For example, the yellow flesh cultivars “Jintao” is a tetraploid, which is a Chinese selection from wild resources and is widely planted in Europe, South America, and China. Moreover, two genetic clusters were revealed in the *A. chinensis* populations, suggesting that intraspecies crosses using the individuals from each of these clusters would be useful in cultivar development of kiwifruit.

## 4. Materials and Methods

### 4.1. Sample Collection

We obtained 263 *A. chinensis* individuals from the National *Actinidia* Germplasm Repository of China, which were collected from 24 wild populations (Figure 3a, Table S4) in 2014–2019. The germplasm samples consisted of 43 diploids and 220 tetraploids. The ploidy levels of most samples were determined in previous studies [22] except 15 individuals from TS, which were determined in this study (Figure S2) using a flow cytometric measurement (FCM) with a CyFlow Ploidy Analyser (Partec, Munich, Germany), as per the protocol in Li et al. [37].

### 4.2. DNA Extraction and Microsatellite Genotyping

Total genomic DNA was extracted from the silica-gel-dried leaves with the cetyltrimethylammonium bromide (CTAB) method [38]. The polyphenols and polysaccharides in kiwifruit leaves were removed at the beginning of extraction. A NanoDrop 8000 spectrophotometer (Thermo Fisher Scientific, Waltham, MA, USA) and 1% agarose gels were used to detect the concentration and quality of the DNA extracted. To assess nuclear DNA polymorphism, 263 individuals were genotyped at 40 nuclear microsatellite loci. These microsatellite polymorphic primer pairs used were a subset of those from Huang et al. [39]. All forward primers were labeled with four kinds of 5′-fluorescein bases (FAM, HEX, TRAMA, or ROX). PCR amplification followed the protocol derived from Huang et al. [39]. Fluorescent-labeled PCR products were supplemented with the internal size standard GeneScan 500 LIZ and separated on a 3730xl DNA Analyzer (Applied Biosystems, Waltham, MA, USA). The detection bands of 40 markers were scored with Genemapper version 4.1. Microsatellite quality was checked for the presence of scoring errors, and large allele dropout was examined with MSAnalyser.

Genotyping microsatellite data corresponding to polyploids can be problematic because of the difficulties in assigning the correct allele dosage for each locus and individual [40,41,42]. In addition, it was also difficult to estimate the allele copy number in tetraploids based on electropherogram peak height, as in Esselink et al. [41,43]. Thus, we created two different format datasets for the next analysis. For those analyses (GENODIVE, POLYGENE, and STRUCTURE) which allow codominant data or ambiguous genotypes, we used genotypic data that were exported from Geneious in the GeneMapper format. Others used the ‘marker phenotypes’ or ‘allelic phenotypes’ dataset which was a binary matrix created by recording the presence (1) or absence (0) of alleles for each microsatellite locus per accession [44,45,46].

### 4.3. SSR Data Analysis

The standard population genetic diversity statistics (such as *F*_ST_) in this study could not be calculated using traditional analytics software [41] because of the tetraploid nature of most *A. chinensis* samples and the dosage effect of polyploid alleles. Therefore, we used POLYSAT 1.5-0 [47] and GENODIVE version 3.04 [44], which can handle genetic data from polyploids or mixed-ploidy datasets and corrects for the unknown dosage of alleles in partial heterozygotes.

Genetic diversity was evaluated through the following descriptive statistics: the number of alleles (*N*a), effective number of alleles (*N*e), and observed (*H*o) and expected (*H*e) heterozygosity and inbreeding coefficient (*F*is), all of which were calculated with GENODIVE. As software developed specifically for the analysis of polyploid genetic data, POLYGENE v1.2 can take into account both polyploid genotypic ambiguities and double reduction [48] and can also infer possible genotypes and their posterior probabilities based on allelic phenotype and inheritance models. To take advantage of these benefits, the observed (*H*o) and expected (*H*e) heterozygosity, polymorphic information content (PIC), and Shannon diversity index (I) were also estimated for each population and locus in POLYGENE v1.2. Differentiation among *A. chinensis* populations was assessed with *G*_ST_ [49]. In addition, to investigate the extent of genetic differentiation among *A. chinensis* populations, analysis of molecular variance (AMOVA) [50] was implemented in POLYGENE. We used AMOVA to examine genetic variation among populations, ploidy, and the two groups separately. To assess the effect of geographic conditions on genetic divergence, the isolation by distance (IBD) was tested with a Mantel test of 10,000 permutations to detect the relationship between geographic distance and genetic distance among populations. To accommodate the existence of polyploid populations, Slatkin’s linearized *F*_ST_ was adopted as the measure of genetic distance [51]. Principal coordinate analysis (PCoA) was performed with the Cavalli-Sforza (1967) chordal distance [52]. Previous studies have shown that in the absence of dose information, principal coordinate analysis is the distance measure with the least bias [46].

To reveal the number of clusters, a Bayesian analysis under an admixture model with correlated allele frequencies was performed with the program STRUCTURE 2.3.4 [53,54]. The potential number of genetic clusters (K) varied from 1 to 20. Ten independent simulations were run for each value of K with 100,000 burn-in steps followed by 1,000,000 Markov chain Monte Carlo (MCMC) steps. The optimum *K* was inferred with the online program STRUCTURE HARVESTER [55,56]. The program CLUMPP v1.1.2 [57] was used to permute the independent replicates for the optimum value of K. The final bar and pie charts for the populations was plotted with District v1.1 [58] and ArcMap v10.3 (ESRI, Redlands, CA, USA). To evaluate genetic relationships, a neighbor-joining tree based on *D*_A_ genetic distance was established for *A. chinensis* populations with POPTREE v.2 [59].

### 4.4. Species Distribution Models (SDMs)

Ecological niche modeling (ENM) was used to predict suitable current and future distribution ranges of tetraploid *A. chinensis* with Maxent v.3.4.0 [60,61]. The present geographic distribution of tetraploid *A. chinensis* was represented by 52 data points extracted from previous studies [22]. In addition, six bioclimatic parameters were identified for ENM, which were the same variables used in a previous study [22]. These parameters with identical spatial resolution were downloaded from the WorldClim Databases (http://www.worldclim.org/, accessed on 1 December 2021) [62]. The future data (i.e., 2041–2060 and 2081–2100) were downloaded from the BCC-CSM2-MR climate change modeling data under the shared socio-economic pathway (SSP). The 1–2.6, 2–4.5, 3–7.0, and 5–8.5 scenarios will be ultimately released by IPCC Assessment Report 6 (AR6). Unlike representative concentration pathways (RCPs), SSPs take into account the socioeconomic and land use impacts on the development of regional climate change when projecting greenhouse gas (GHG) emission scenarios for different climate policies in the future [63]. In this study, the model quality was assessed with cross-validation comprising 10 replicates with 75% of the data for model training and 25% of the data for model testing. The maximum number of background points was 10,000. To calibrate the model goodness of fit, the area under the receiver operating characteristics curve (AUC) was examined to verify the model precision [64]. For further analysis, the result of ENM was imported into ArcGIS 10.3 (ESRI) and classified as four possible habitat types, including “not” (<0.1), “hardly” (0.1–0.35), “moderately” (0.35–0.65), and “highly” (>0.65) suitable habitats.

## 5. Conclusions

This study revealed the genetic diversity and structure of 19 tetraploid populations in *A. chinensis*. It also compared the genetic diversity and structure between two cytotypes within the *A. chinensis*. In addition, changes in potentially suitable regions for tetraploid *A. chinensis* were modeled with ENM. Based on the results of our analyses, considerable levels of genetic diversity exist among tetraploids in *A. chinensis*, and its potential suitable area will likely be reduced in the future. These results can serve as basic information by providing options to breeders to develop, through selection and breeding, new and more productive varieties that are adapted to changing environments. In addition, this will also provide a reference basis for the protection of wild tetraploid *A. chinensis* resources.

## Figures and Tables

**Table 1:** Genetic diversity of 24 *A. chinensis* populations based on 40 SSR markers.

Population	GENODIVE					POLYGENE			
	*N*a	*N*e	*H*o	*H*e	*F*is	*H*o	*H*e	PIC	I
GT	5.050	3.709	0.552	0.756	0.269	0.738	0.684	0.648	1.432
NL	4.625	3.109	0.537	0.670	0.198	0.681	0.636	0.598	1.294
ZG	4.250	3.037	0.598	0.672	0.109	0.721	0.636	0.593	1.25
NC	6.400	4.519	0.648	0.787	0.178	0.803	0.746	0.714	1.64
LA	7.525	5.289	0.737	0.802	0.080	0.808	0.761	0.736	1.749
JX	10.250	5.842	0.625	0.805	0.224	0.779	0.775	0.756	1.925
QM	7.750	4.901	0.616	0.829	0.256	0.77	0.763	0.743	1.796
XN	9.675	5.602	0.627	0.803	0.219	0.779	0.772	0.752	1.902
PC	7.000	4.384	0.528	0.811	0.349	0.738	0.735	0.709	1.675
PN	6.675	3.863	0.566	0.698	0.189	0.69	0.682	0.649	1.515
SW	7.575	4.295	0.571	0.770	0.258	0.733	0.721	0.698	1.675
ZY	12.775	6.437	0.534	0.800	0.333	0.755	0.748	0.73	1.908
TS	12.025	6.296	0.594	0.817	0.272	0.78	0.776	0.759	1.99
DN	8.100	5.252	0.522	0.855	0.389	0.75	0.737	0.713	1.738
HJ	11.025	6.118	0.584	0.809	0.278	0.775	0.773	0.754	1.946
LS	10.825	6.092	0.604	0.809	0.253	0.79	0.786	0.766	1.968
TG	9.975	5.446	0.625	0.804	0.223	0.785	0.776	0.757	1.908
WN	10.425	5.772	0.641	0.799	0.197	0.79	0.788	0.768	1.947
ZX	9.475	5.035	0.552	0.782	0.294	0.749	0.741	0.719	1.785
JD	11.375	6.354	0.621	0.822	0.244	0.8	0.795	0.777	2.015
LD	9.850	5.774	0.563	0.816	0.310	0.77	0.763	0.741	1.864
SC	9.150	5.187	0.611	0.801	0.237	0.778	0.778	0.755	1.873
TT	8.800	5.225	0.610	0.804	0.241	0.771	0.77	0.747	1.85
ZJ	9.700	5.434	0.650	0.805	0.193	0.791	0.78	0.759	1.89
Average (2x)	5.570	3.933	0.614	0.737	0.167	0.75	0.693	0.658	1.473
Average (4x)	9.601	5.437	0.592	0.802	0.265	0.767	0.761	0.736	1.851
Average (all)	8.761	5.124	0.597	0.789	0.241	0.764	0.746	0.723	1.772

*N*a: number of alleles for each population; *N*e: effective number of alleles for each population; *H*o: observed heterozygosity; *H*e: expected heterozygosity; *F*is: inbreeding coefficient; I: Shannon’s Information Index; PIC: polymorphic information content.

**Table 2:** Analysis of molecular variance (AMOVA) for *A. chinensis* populations.

Source of Variation	DF	SS	MS	Variance Component	Percentage of Variation (%)	Fixation Index
Among populations	23	2829.428	123.019	5.865	9.01%	FST = 0.090 *
Within populations	239	14,154.572	59.224	59.224	90.99%	
Among clusters	1	279.915	279.915	1.642	2.50%	FST = 0.025 *
Within clusters	261	16,704.085	64.000	64.000	97.50%	
Among ploidy types	1	215.479	215.479	2.102	3.17%	FST = 0.032 *
Within ploidy types	261	16,768.521	64.247	64.247	96.83%	

*: *p* < 0.001, *p* values based on 10,000 permutations.

## Data Availability

Data are contained within the article.

## Supplementary Materials

The following supporting information can be downloaded at: https://www.mdpi.com/article/10.3390/plants11091154/s1, Figure S1: Principal coordinate analysis (PCoA) of *A. chinensis* based on 40 SSR markers; Figure S2: Schematic diagram of flow cytometric histogram of DAPI-stained nuclei of tetraploid *A. chinensis* (4x) analyzed simultaneously with the internal standard *Actinidia chinensis* cv. ‘Hongyang’ (2x). Table S1: Summary statistics for the 40 SSR markers across all *A. chinensis* populations with metrics of genetic diversity estimated using GENODIVE 3.04 and POLYGENE version 1.2b.; Table S2: 758 SSR alleles of 263 *A. chinensis* individuals sampled from 24 populations; Table S3: Pairwise divergence among *A. chinensis* populations based on Nei’s *G*_ST_; Table S4: Sampling information for *A. chinensis* populations.
