# Supplementary material for: Characterizing Tetraploid Populations of Actinidia chinensis for Kiwifruit Genetic Improvement
Source: Plants (Basel). 2022 Apr 24;11(9):1154. doi: 10.3390/plants11091154 (PMC9102457; doi:10.3390/plants11091154)
Supplement: Supplementary file 1 [file plants-11-01154-s001.zip › Figure S1-2.pdf]

Supplementary Flies

Characterizing tetraploid populations of *Actinidia chinensis* for kiwifruit genetic improvement

Zhi Wang [1,2], Guangming Hu [2,3], Zuozhou Li [2], Caihong Zhong [2,*] and Xiaohong Yao [2,*]

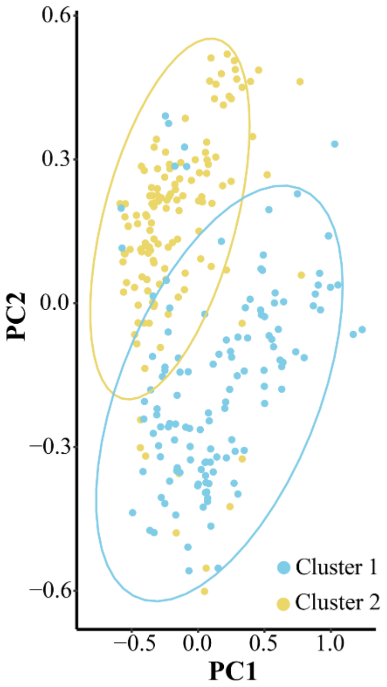

Figure S1. Principal coordinate analysis (PCoA) of A. *chinensis* based on 40 SSR markers.

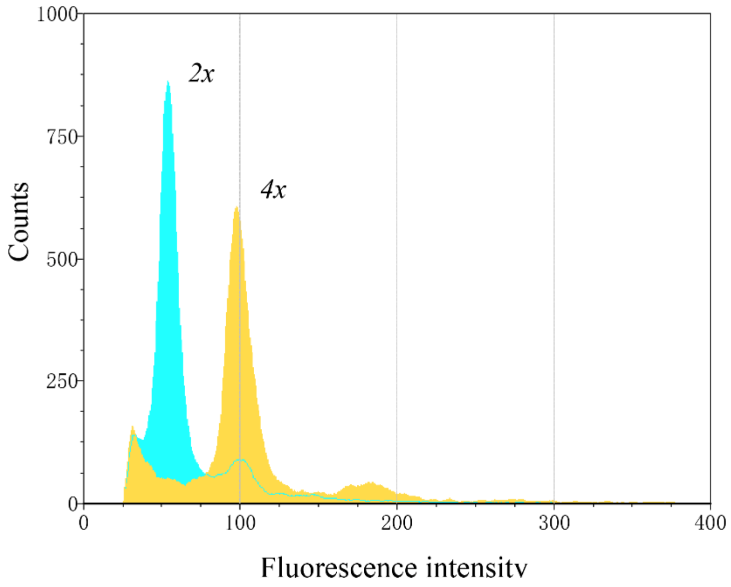

Figure S2. Schematic diagram of flow cytometric histogram of DAPI-stained nuclei of tetraploid *A. chinensis* (4x) analyzed simultaneously with the internal standard *Actinidia chinensis* cv. 'Hongyang' (2x).
